# Investigation on *Mycobacterium tuberculosis* Diversity in China and the Origin of the Beijing Clade

**DOI:** 10.1371/journal.pone.0029190

**Published:** 2011-12-29

**Authors:** Kanglin Wan, Jinghua Liu, Yolande Hauck, Yuanyuan Zhang, Jie Liu, Xiuqin Zhao, Zhiguang Liu, Bing Lu, Haiyan Dong, Yi Jiang, Kristin Kremer, Gilles Vergnaud, Dick van Soolingen, Christine Pourcel

**Affiliations:** 1 Univ Paris-Sud, Institut de Génétique et Microbiologie, UMR 8621, Orsay, France; 2 CNRS, Orsay, France; 3 National Institute for Communicable Disease Control and Prevention, Chinese Center for Disease Control and Prevention/State Key Laboratory for Infectious Disease Prevention and Control, Beijing, People's Republic of China; 4 National Mycobacteria Reference Laboratory, National Institute for Public Health and the Environment (RIVM), Bilthoven, The Netherlands; 5 Departments of Clinical Microbiology and Pulmonary Diseases, Radboud University Nijmegen Medical Center, Nijmegen, The Netherlands; 6 DGA/MRIS- Mission pour la Recherche et l'Innovation Scientifique, Bagneux, France; St. Petersburg Pasteur Institute, Russian Federation

## Abstract

**Background:**

Investigation of the genetic diversity of *Mycobacterium tuberculosis* in China has shown that Beijing genotype strains play a dominant role in the tuberculosis (TB) epidemic. In order to examine the strain diversity in the whole country, and to study the evolutionary development of Beijing strains, we sought to genotype a large collection of isolates using different methods.

**Methodology/Principal Findings:**

We applied a 15-loci VNTR typing analysis on 1,586 isolates from the Beijing municipality and 12 Chinese provinces or autonomous regions. The data was compared to that of 900 isolates from various other worldwide geographic regions outside of China. A total of 1,162/1,586 (73.2%) of the isolates, distributed into 472 VNTR types, were found to belong to the Beijing genotype family and this represented 56 to 94% of the isolates in each of the localizations. VNTR typing revealed that the majority of the non-Beijing isolates fall into two genotype families, which represented 17% of the total number of isolates, and seem largely restricted to China. A small number of East African Indian genotype strains was also observed in this collection. Ancient Beijing strains with an intact region of difference (RD) 181, as well as strains presumably resembling ancestors of the whole Beijing genotype family, were mainly found in the Guangxi autonomous region.

**Conclusions/Significance:**

This is the largest *M. tuberculosis* VNTR-based genotyping study performed in China to date. The high percentage of Beijing isolates in the whole country and the presence in the South of strains representing early branching points may be an indication that the Beijing lineage originated from China, probably in the Guangxi region. Two modern lineages are shown here to represent the majority of non-Beijing Chinese isolates. The observed geographic distribution of the different lineages within China suggests that natural frontiers are major factors in their diffusion.

## Introduction

Tuberculosis (TB) affects millions of people worldwide with an estimated global prevalence of 164 per 100,000 population. Although the incidence is believed to be generally slowly declining, this disease remains a major health problem in many countries. The average prevalence of TB in China amounts to 367 per 100,000 and this country has the highest absolute number of cases annually in the world. Among TB patients notified in China in 2009, slightly more than 30,000 (12%) were diagnosed and notified as multidrug resistant TB (MDR-TB) but this number may be underestimated [1]. BCG vaccine is not providing sufficient protection against tuberculosis and breakdown to disease, and this may in particular favor the emergence of new genotypes with enhanced virulence, such as the Beijing genotype, as shown in animal models and recently in humans in Vietnam [2], [3], [4].

To investigate the population structure of *M. tuberculosis* and define genetic lineages, several methods have been developed including spoligotyping [5], [6], single nucleotide polymorphisms [7], variable number of tandem repeat (VNTR) [8], [9], [10], [11], large sequence polymorphism (LSP) typing [12], [13], [14], [15], partial [16], [17] or whole genome [18] sequence analysis. Worldwide, nine superfamilies of *M. tuberculosis* strains with a preferred geographic distribution were described using spoligotyping [19]. Further studies based on LSP [12], [15] and on the sequence of 89 genes [16] or whole genome sequencing [18] allowed the definition of 6 lineages globally highly congruent with spoligotyping defined lineages, with some exceptions. Lineage 4 (Euro-American) includes a number of spoligotype patterns which cannot be readily classified based on spoligotyping only. Lineage 2 contains, in addition to the Beijing family, strains with poorly informative spoligotypes [17].

The Beijing family was described for the first time as a genetically closely related genotype family in 1995, one of its characteristics being the absence of spacers 1 to 34 in the direct repeat (DR) locus [6]. In the early 1990 s the ‘W’ strains, later shown to constitute a minor branch of the Beijing family, were associated with the spread of MDR-TB in North American cities [20], [21]. In multiple areas in the world, such as Vietnam, Russia and South Africa the Beijing genotype was correlated with TB in young patients, and, hence, it is thought to be emerging rapidly [22], [23], [24]. Such observations raise the question of the origin of this family. Spoligotyping of *M. tuberculosis* bacteria in paraffin-embedded material from a hospital in Beijing demonstrated that the Beijing strains were already present in China in 1956 [25]. Later studies showed that these strains were mostly prevalent in East Asia [6], [21], [26] and the former Soviet Union [23], [27], [28], [29], but also South Africa [30]. Recent reports also described a high prevalence of Beijing strains in Japan (70–80%) [31], [32], [33], [34] but the largest percentages were observed in China with a prevalence of 93% in the Beijing municipality area [35], [36], [37].

Members of the Beijing genotype family have previously been identified and classified based upon the IS*6110* insertion site (A1) in the origin of chromosome replication (oriC) [38], [39]. Later, Beijing strains were subdivided into modern/Typical and ancient/Atypical sublineages based on the analysis of the NTF-1 locus [29] or IS*6110* insertion profiles [40], [41]. In addition to the characteristic deletion of the DR locus (affected by RD207), Tsolaki *et al*. described LSPs (RD105, RD142, RD150, and RD181) which further divided this family into monophyletic subgroups [42], the more ancestral event being the RD181 deletion. Some studies have shown that not all ancient/Atypical strains are RD181 intact (RD181 [+]) which indicates that the RD181 deletion occurred before the insertion of an IS element in the NTF-1 locus [34], [43]. RD105 was deleted in all Beijing genotype strains but also in RD207 [+] strains belonging to lineage 2 [12], [17]. It has been suggested that the Beijing family successfully expanded relatively recently from a single ancestor which presumably had selective advantages over other genotypes of *M. tuberculosis* [6], [44], [45]. Demographic factors may also be responsible for this predominance in particular geographic areas. Besides, the relative homogeneity of Asian populations at the anthropological level may contribute to the genetic conservation of *M. tuberculosis* strains in China, owing to co-evolution between the host and the pathogen [46], [47]. Phylogeographic studies that considered the population structures of Beijing genotype strains and humans suggested that the Beijing lineage originated in Central Asia. However these studies were based on relatively small numbers of isolates from Chinese in Singapore [29].

To further explore the population structure of *M. tuberculosis* in China, geographically large surveys still need to be performed with a combination of genotyping approaches. VNTR typing is increasingly being used as a first line assay owing to its discriminatory power, to the relevance of the clustering it achieves, and to the availability of relatively large data sets [11]. Although a 24-VNTR loci protocol has been proposed to be the reference in standard typing, different combinations of VNTRs are used alone, or with spoligotyping or IS*6110* restriction fragment length polymorphism (RFLP) [28], [48], [49], [50], [51], [52], [53], [54]. When an appropriate collection of markers is applied, VNTR typing recognizes the major genotype families within the *M. tuberculosis* complex [17], [45]. To obtain sufficiently informative analysis and robust clustering, it is necessary to use a panel of markers with different discrimination indexes and to combine the data with other typing approaches [17], [55], [56]. In the present survey involving 1,586 Chinese isolates among 2,346 previously analyzed by spoligotyping [37], we aimed to be able to describe unrecognized families and to search for potential ancestors of the Beijing genotype family.

## Results

### *M. tuberculosis* diversity in China

Spoligotyping was not sufficiently discriminative for in depth analysis of the *M. tuberculosis* population in China [37] and we therefore decided to apply the VNTR genotyping technique, which provides a higher level of phylogenetic information [17]. In order to facilitate efficient genotyping of large number of isolates we selected a limited panel of highly typable VNTRs that would correctly cluster the bacteria into the main clades/genotype families. A first series of 98 isolates from the Beijing municipality and 4 different provinces were genotyped with 21 VNTRs (VNTR21_Orsay_) as previously described [10]. Clustering was performed with different combinations of VNTRs and we retained a set that was amenable to easy manual reading of agarose gel images while producing a sufficient degree of information (Figure S1 and Table S1). We furthermore eliminated markers that yielded technical problems in the amplification or visual gel image analysis such as Mtub02 (9 bp repeat) or that were unstable such as Qub11a, or were not very informative such as Mtub12. The selected 15 VNTR loci forming the VNTR15_China_ scheme were: ETR-A, ETR-B, ETR-C, ETR-D (alias MIRU04), ETR-E (alias MIRU31), MIRU10, MIRU16, MIRU23, MIRU26, MIRU27, MIRU39, MIRU40, Mtub21, Mtub30, Mtub39. The 15 VNTRs of this scheme are included in the VNTR24 scheme described by Supply et al. [11]. Eleven are shared with the VNTR15 scheme [11], and nine are in common with the earlier MIRU12 selection of loci [57]. We retained ETR-B (VNTR 2461), MIRU23 (VNTR 2531), MIRU27 (VNTR 3007) and MIRU39 (VNTR 4348) which present a lower level of allelic diversity but are useful to anchor the different lineages [17] and to make comparisons with published data sets. Thereafter VNTR15_China_ was performed on all the collected isolates from nine regions and only the non-Beijing isolates for the Beijing municipality and the Fujian province. The total number of samples for which data were obtained was 1,586, of which 1,162 belonged to the Beijing family and 424 were non-Beijing according to the spoligotyping (Table 1 and Table S2) [37].

**Table 1:** Number of isolates genotyped by VNTR21_Orsay_ or VNTR15_China_ in each province.

Origin^a^	Beijing^b^	non-Beijing	Total
(21 VNTRs)			
Anhui (AH)	24	6	30
Beijing (BJ)	19	0	19
Fujian (FJ)	12	9	21
Hunan (HN)	14	4	18
Jiangsu (JS)	8	2	10
Total	77	21	98
(15 VNTRs)			
Beijing (BJ)	0	8	8
Fujian (FJ)	0	44	44
Gansu (GS)	58	14	72
Guangxi (GX)	113	89	202
Hunan (HN)	64	27	91
Jilin (JL)	288	38	326
Shanxi (ShX)	78	16	94
Sichuan (SC)	65	37	102
Xinjiang (XJ)	134	61	195
Xizang (XZ)	173	18	191
Zhejiang (ZJ)	112	51	163
Total	1085	403	1488
All isolates	1162	424	1586

a includes municipality, provinces and autonomous regions.

b based on spoligotyping.

Clustering analyses were performed by UPGMA using the categorical coefficient, and 17 groups differing by a maximum of five VNTRs (cut-off value of 60%) were defined. The larger cluster corresponded to the Beijing isolates as confirmed by spoligotyping and three clusters showed the signature of isolates belonging to lineage 4 (which includes all spoligotype clades with deletion of spacers S33 to S36). Other clusters showed mutually recognisable spoligotype signatures. For 20 strains there was no concordance with the spoligotyping results. In particular, ten isolates with a spoligotype profile corresponding to Manu2 (absence of S33 and S34 [58]) were clustered with Beijing strains or with lineage 4 strains. These confusing findings suggest a superposition of both a Beijing and a lineage 4 profile as might result from mixed strains and were therefore excluded from further analysis.

**Figure 1:**
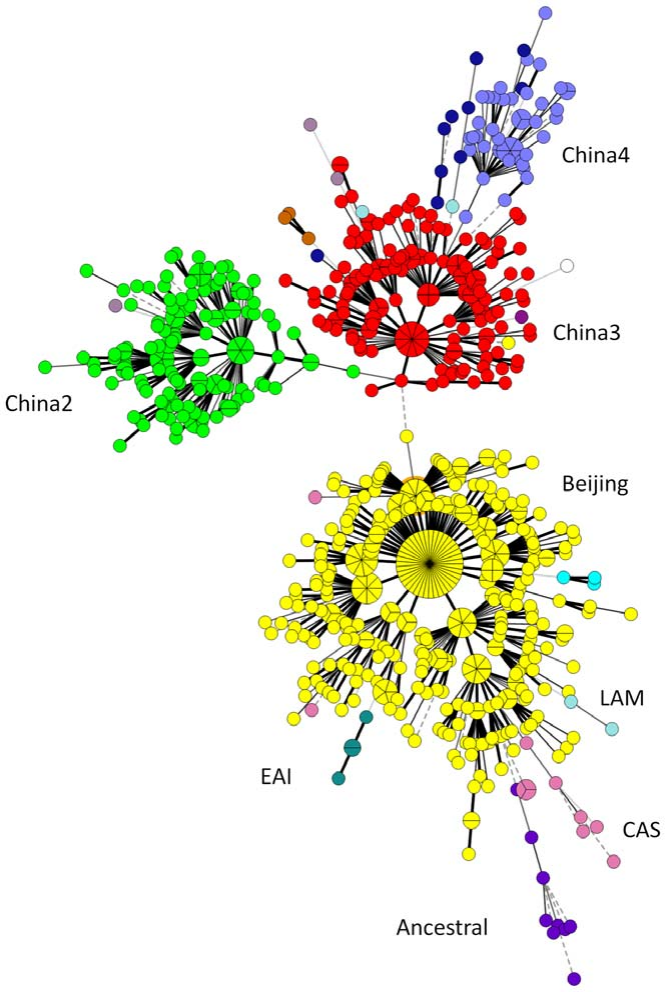
Minimum spanning tree showing the clustering by MLVA15_China_ of 805 *M. tuberculosis* isolates comprising 401 Beijing isolates from Jilin and Guangxi provinces, and 404 non-Beijing isolates. The correspondence with clades defined by spoligotyping is indicated near each coloured cluster.

In Figure 1 a minimum spanning tree shows the clustering of 401 Beijing isolates originating from the Jilin province and Guangxi autonomous region and of the 404 non-Beijing isolates (Table 1: the remaining Beijing isolates were not included to simplify the figure). Three clusters shown in green, red and blue (respectively indicated as China2, China3 and China4) had the lineage 4 spoligotype signature (absence of S33 to 36). The pink cluster corresponded to lineage 3 (CAS) strains, and the purple one may represent an ‘ancestral’ lineage or sub-lineage.

To further strengthen the assignation of isolates to known *M. tuberculosis* clades, the genotypes of Chinese isolates were compared to those of a large collection of isolates from other countries worldwide (616 from our own database in Orsay, Table S3 which can also be queried at http://mlva.u-psud.fr; and 186 from the VNTRplus database held at http://www.miru-vntrplus.org/) in which the major clades of the *M. tuberculosis* complex are represented. The nomenclature of the spolDB4 and of the MIRU-VNTR database was used to identify the clusters. The result indicated that China2 is not represented in the reference data set used here, and that China3 seemed to be almost entirely restricted to China (Figure S2). These genotypes accounted for 17% of the total Chinese collection of isolates (Table 2). In contrast China4 is found worldwide.

**Table 2:** Distribution of *M. tuberculosis* China 2 and China 3 isolates in nine regions.

Origin^a^	Nbr of isolates	China 2 isolates	Rate (%)	China 3 isolates	Rate (%)
Gansu	72	2	2.77	4	5.55
Guangxi	202	25	12.3	33	16.3
Hunan	91	12	13.18	11	12.08
Jilin	326	15	4.60	15	4.60
Shanxi	94	4	4.25	8	8.51
Sichuan	102	19	18.63	13	12.74
Xinjiang	195	12	6.15	23	11.79
Xizang	191	5	2.61	8	4.18
Zhejiang	163	20	12.27	19	11.65
Total	1436	114	7.93	134	9.33

a The number of isolates from Anhui, Beijing, Fujian and Jiangsu was too low to be included in this table.

### Distribution of clades in the different Chinese regions

Clustering of VNTR15_China_ typing data of all isolates was performed for each location separately in order to better evaluate the importance of the major clades in the collection and to show the occurrence of the newly identified groupings (Table 2). The distribution of isolates in Xinjiang and Guangxi is shown on Figure S3 and Figure S4, respectively. A group of isolates specific for Xinjiang in the present investigation is circled in Figure S3. The Guangxi autonomous region possesses the lowest percentage of Beijing-family strains and a larger diversity in non-Beijing strains. The analysis revealed the existence of a group of seven isolates, clustering together with the Beijing and lineage 3 (CAS) strains (purple in Figure 1 and circled in Figure S4). Table 3 shows the spoligotypes of all the isolates from this group, as well as one from Sichuan and one from Zhejiang. An analysis of different regions of genomic deletions was performed showing that TbD1 and RD105 were deleted in all isolates of this grouping (data not shown). This is the signature of lineage 2 strains. A preliminary study of the DR locus by PCR revealed the presence of an additional group of spacers not detected by spoligotyping, and which is characteristic of Beijing family strains (spacers 48 to 50) [59]). These characteristics strongly suggest that this group of 9 strains, 7 of which were found in the Guangxi region, might represent the ‘ancestor’ of the whole Beijing family. Nine strains from Xinjiang (Figure S3) and three isolates from Tibet showed a typical lineage 3 (CAS) spoligotype pattern and clustered by VNTR typing with lineage 3 CAS-Delhi isolates from other geographical origins (Figures 1 and S2). Isolates with a typical lineage 1 (EAI) spoligotype signature (deletion of spacer 29–32 and 34) were found only in Fujian province and were distributed into two clusters.

**Table 3:** Spoligotype of ancestral strains.

Strain	Spoligoprofile^a^	SIT^b^
GX06002	1111111111111111111111111111111101111110111	
GX06030	1111111111111111111111111111111111111110111	246
GX06040	1111111111111111111111111111111111111110111	246
GX06121	1111111111111111111111111111111111101111111	
GX06145	1111111111111111011000001011111111111110111	
GX06162	1111111111111111111111111111111111111111111	523
GX06203	0111111111111111111111111111111111111110111	
ZJ06098	1111111111111111111111011111111111111111111	623
SC06005	1111111111111111111111111111111111111111111	523

a : 1, presence of the spacer; 0, absence of the spacer.

b : SIT correspond to the Spoldb4 international database code accessible at http://www.pasteur-guadeloupe.fr/tb/bd_myco.html.

Strains XJ06036 and XJ06002 clustered with LAM isolates (members of lineage 4). One strain from Jilin province clustered with *M. bovis*.

### The origin of the Beijing clade

**Figure 2:**
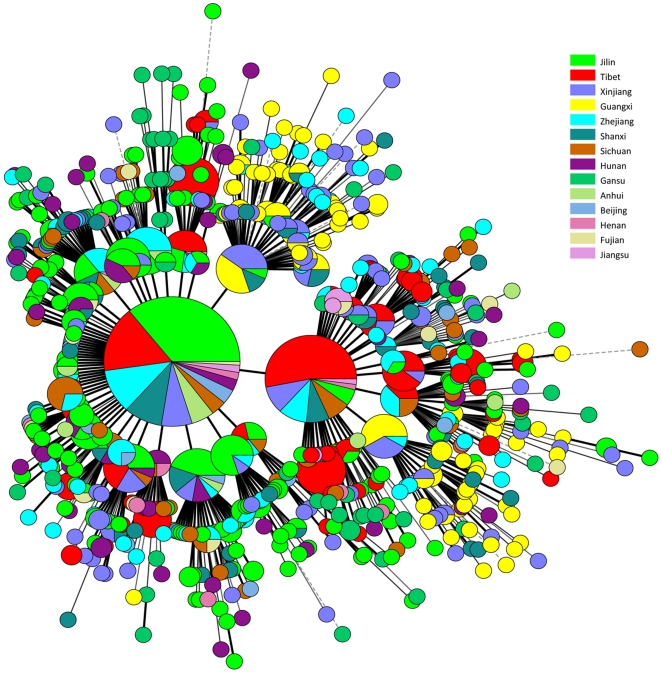
Diversity of *M. tuberculosis* Beijing family in China. Each region is assigned a colour. Isolates of the Guangxi autonomous region, coloured in yellow, are mostly distributed into two clusters. The colour code is indicated on the side.

The 1,162 Beijing isolates genotyped in this study represented 56 to 94% of the isolates in the different parts of China. Only 21 spoligotypes were observed among these isolates, whereas VNTR15_China_ typing identified 472 genotypes. Two hundred and eleven clusters were observed when a cut-off value of 90% was used (1–2 alleles differences between genotypes). One genotype accounted for 16% of the isolates (183) from all origins, except for the Guangxi autonomous region. Figure 2 shows the genotypes distribution in the different regions using different colors. Interestingly, the isolates from Guangxi autonomous region shown in yellow are mostly found in two clusters. This may be explained by transmission and clonal expansion together with a lower degree of mobility of people in this province as reported in the “2011 Report on China's Migrant Population Development” (July 2011). In addition the Guangxi and Xinjiang regions show similar distributions.

We furthermore tried to identify the more ancient RD181 [+] strains of the Beijing family, in order to investigate the geographical origin and possible source of this important genotype family. For this we tested for the presence/absence of the RD181 region by PCR using primers localized outside (external) or inside (internal) this region (data not shown). Data from 1,466 *M. tuberculosis* Beijing family strains identified by spoligotyping from 12 locations were used in the present study. The percentage of RD181 [+] strains varied from 3.3% to 16.5% as shown in Table 4. Guangxi, in which the highest percentage of such strains was found, is also the province with the lowest percentage of Beijing strains (55%).

**Table 4:** Percentage of Beijing family and RD181 [+] isolates in 12 regions.

No	Province	No. of Beijing family isolates^a^	RD181 [−]	RD181 [+]
		(%)	(%)	(%)
1	Beijing	113 (93.4)	103 (93.6)	7 (6.4)
2	Fujian	100 (56.2)	82 (91.1)	8 (8.9)
3	Gansu	149 (85.1)	122 (89.7)	14 (10.3)
4	Guangxi	115 (55.3)	96 (83.5)	19 (16.5)
5	Henan	67 (83.8)	58 (87.9)	8 (12.1)
6	Hunan	68 (70.1)	58 (96.7)	2 (3.3)
7	Jilin	298 (89.5)	252 (85.1)	44 (14.9)
8	Shanxi	97 (80.8)	75 (89.3)	9 (10.7)
9	Sichuan	66 (61.7)	60 (93.7)	4 (6.3)
10	Xinjiang	135 (66.2)	104 (89.7)	12 (10.3)
11	Xizang	194 (90.2)	179 (95.7)	8 (4.3)
12	Zhejiang	64 (64.6)	63 (98.3)	1 (1.7)
	Total	1,466 (75.68)	1,073 (88.8)	136 (11.2)

a not all isolates were genotyped using VNTRs. For this reason values indicated in Table 1 are lower.

## Discussion

### A survey on the strain diversity in Beijing municipality and 12 provinces and autonomous regions

This is the first extended, detailed study on the genetic diversity of *M. tuberculosis* covering a significant part of China using several genotyping approaches. It describes not only the population structure of *M. tuberculosis*, including the presence of genotype families not or rarely found elsewhere in the world, but also provides information on the possible origin of Beijing genotype strains. There was an obvious genetic diversity in the *M. tuberculosis* strains isolated from the different parts of China, although the main epidemic strain cluster in the different locations is formed by the lineage 2 Beijing family (RD105 [−] and RD207 [−]) isolates.

**Figure 3:**
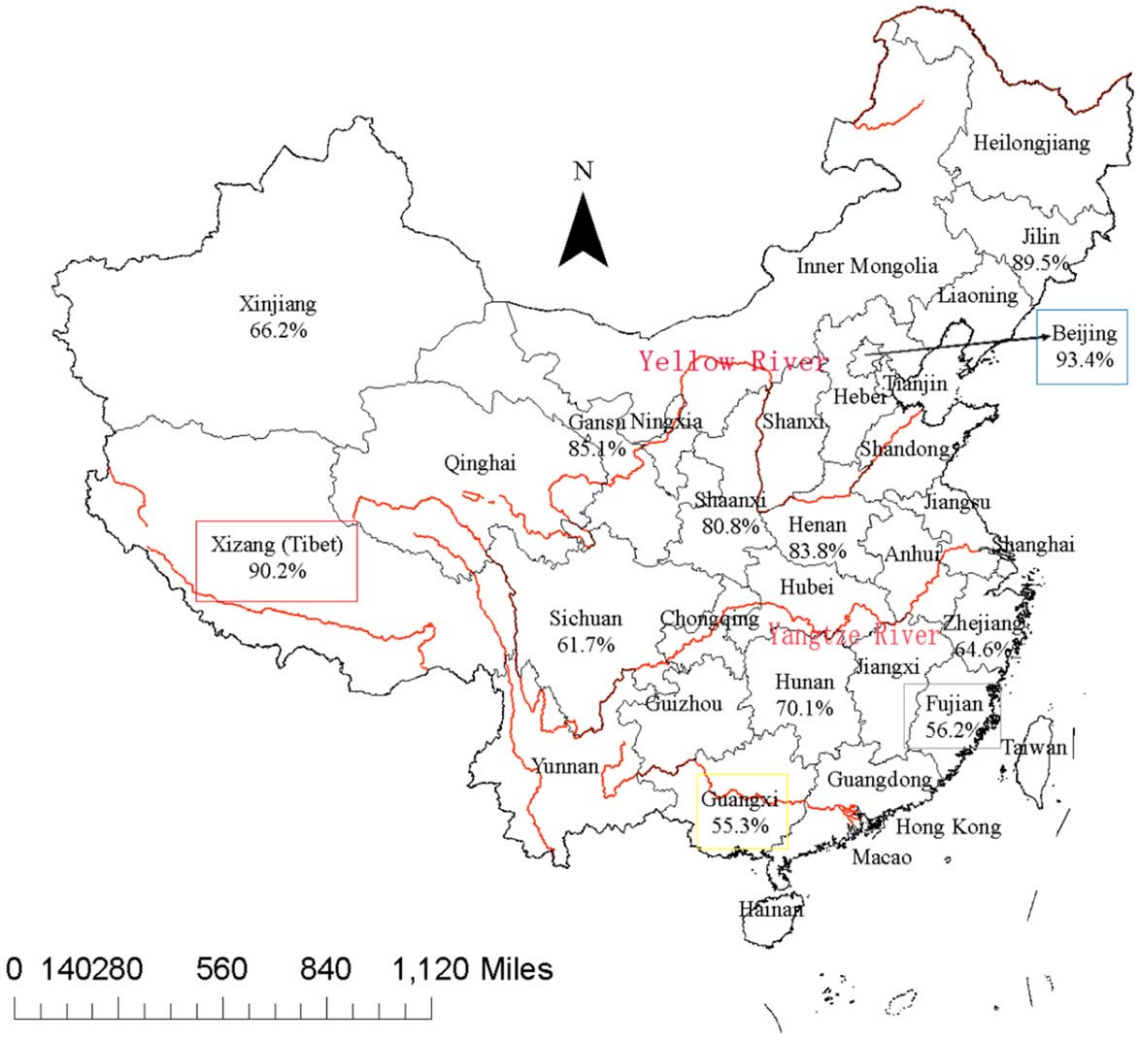
Map of China showing the proportion of Beijing-family strains in the investigated regions.

As previously shown by spoligotyping performed on the same samples [37], the highest density of Beijing strains was observed in the Northern and Western part of China, including Xizang (Tibet) with the exception of Xinjiang autonomous region which is mostly populated by Uyghurs. The region with the second highest prevalence of Beijing strains was the central part of China, e. g. between Yellow River and Yangtze River, and the lowest was observed in southern China (Figure 3). The VNTR typing added to the spoligotyping results revealed a high degree of polymorphism in this family. The second more abundant lineage in China is lineage 4 with three main subgroups of which two are nearly exclusive for China (China2 and China3) according to available VNTR typing data and one is found worldwide (China4). In Xinjiang another lineage 4 subgroup specific for this region was observed. These subgroups could only be revealed by VNTR typing as their spoligotype is not specific [37]. A small group of isolates belonging to lineage 3 (CAS) was observed in Xinjiang and Xizang (Tibet), both close to India where isolates of this family are very abundant. In Xinjiang, three isolates clustered with isolates of the LAM clade, frequently found in Latin American and African countries but also in Russia and Central/Eastern Asian countries [60]. However, the spoligotyping profile is unclassified and does not show the signature of typical LAM isolates (deletion of S21 to S24).

Interestingly, the only isolates belonging to lineage 1 (EAI) were found in Fujian province, although such strains are very abundant in other countries of Eastern Asia, like Vietnam [22]. No isolate clustered by VNTR15_China_ typing with isolates of ancestrally branching-out Manu lineages, although spoligotypes characteristic of these clades could be occasionally observed. The most likely explanation is the presence of mixed infections with Beijing and non-Beijing strains such as previously reported in Taiwan [61]. Indeed superposition of lineage 2 and lineage 4 spoligotypes (the two most frequent lineages in China) would create a typical but artefactual Manu spoligotype (deletion of spacer 33).

### The Beijing family

The results obtained on the basis of VNTR15_China_ typing were in agreement with spoligotyping data. However, although the majority of Beijing genotype strains aggregated into a large homogenous group, some showed more polymorphism as seen for example in the Guangxi autonomous region (Figure S4). It is assumed that the more clonal and more frequent modern/Typical Beijing strains that are emerging in several parts of the world [21], [44] are derived from ancient/Atypical Beijing. Contemporary representatives of these ‘ancient’ lineages show a higher degree of genetic diversity [62]. Interestingly, Guangxi, in which the lowest rate of Beijing strains was found, is also the region where the largest proportion of RD181 [+] Beijing isolates occurred (16.5%, Table 4) and where a relatively large number of ‘ancestral’ RD207 [+] strains were observed. Some of these strains have a complete spoligotype pattern (Table 3 and [63]). They are thought to be representatives of lineages branching out before the emergence of the Beijing genotype family. Flores et al. first showed that a proportion of strains with such an ancestral spoligotype were RD105 [−], and that they originated from East Asia, Vietnam, China and Laos [63]. In Japan a high incidence of Beijing family strains exists with a high level of ancient/Atypical RD181 [+] strains within the Beijing genotype [34], [64], [65], [66]. A proportion of 5.5% Beijing family strains are RD181 [+] on average across Japan [34], and this percentage raises to 10% (48/498) in the Chiba prefecture [66]. Interestingly Kang *et al.* [34], [43] report a remarkable proportion of 45.3% RD181 [+] strains among 64 Beijing family strains from diverse geographic origin in South Korea. Few publications investigate RD105 [−] RD207 [+] isolates, because these strains which belong to lineage 2 and are very close to the Beijing genotype are not readily identified as such by spoligotyping. Yokoyama *et al.* report the presence of 12 RD207 [+] isolates among a total of 510 lineage 2 isolates (2.35%) in the Chiba prefecture [66]. This can be compared to the ratio of 7/115 (6.08%) observed in the Guangxi region.

Based on the observations described in the present work it is tempting to speculate that TB has the longest history in the South part of China and that Beijing strains have emerged from there. Whole genome sequencing of key isolates as identified by the present investigation may reveal whether the ‘ancestral’ RD207 [+] lineages which can be found in the Guangxi province are likely candidates to represent the lineage from which the ancient/Atypical RD207 [−] RD181 [+] and the emerging and more clonal modern/Typical Beijing strains developed. Such an analysis will also facilitate more phylogenetic studies on the genetic relatedness between different *M. tuberculosis* lineages that determine the current TB epidemic worldwide.

Taking all studies on the prevalence of Beijing genotype strain in China together, the conclusion is that there is a significant diversity in clinical *M. tuberculosis* isolates from China. Beijing family strains, representing 56 to 94% of the isolates in each of the 12 studied regions, is the main prevalent genotype. The subgroups of lineage 4 of which two are mainly found in China (China2 and China3) might be emerging and deserve specific attention as they might possess particular characteristics. Some strains, presumably representing ancestors of the whole Beijing genotype family, were found mainly in Guangxi autonomous region. Further studies on the composition of the genome of these strains and of those in other regions of the world should give clues about their origin and about the mechanisms underlying the enhanced capacity to gain resistance and restore fitness recently acquired by the Beijing sublineage.

It is hoped that the snapshot of the *M. tuberculosis* diversity in China as investigated here will serve as a reference for future investigations, and help evaluate the temporal and geographic dynamics of the emergence and disappearance of lineages in China.

## Materials and Methods

### Ethics statement

The study obtained approval from the Ethics Committee of National Institute for Communicable Disease Control and Prevention, Chinese Center for Disease Control and Prevention. The patients with TB included in the present research protocol were given a Subject information sheet and they all gave written informed consent to participate in the study.

### Collection and identification of clinical isolates in China

From the period 2005 to 2007, 2,346 *M. tuberculosis* isolates were randomly collected from sputum samples of TB confirmed patients in institutes for TB control and cure, as well as TB hospitals distributed in each region included in the study [37]. We tried to equally divide the collected isolates over both sexes and from different age categories (although patients aged 0–16 were underrepresented). The total number of isolates when the collect ended was different in the different regions (Table 1). All isolates were analyzed by spoligotyping and a subset of 1,586 *M. tuberculosis* clinical strains were retained for more detailed molecular typing. The isolates studied in the present work were from the Beijing municipality (BJ) and the following 12 different provinces or autonomous regions: Anhui (AH), Fujian (FJ), Gansu (GS), Guangxi (GX), Hunan (HN), Jiangsu (JS), Jilin (JL), Shanxi (ShX), Sichuan (SC), Xinjiang (XJ), Xizang/Tibet (XZ), Zhejiang (ZJ). Only few isolates could be recovered from the Anhui and Jiangsu province during the time of this investigation. The strain identity code starts with the indicated initials.

The patients were diagnosed on the basis of a Ziehl-Neelsen smear-positive sputum and/or showed signs of pulmonary tuberculosis on X-ray. *M. tuberculosis* was cultured on Lowenstein-Jensen or Coletsos medium. Growing acid fast bacilli were identified according to conventional biochemical procedures (PNB/TCH differential medium) and growth characteristics, and the drug susceptibility was assessed by the proportional drug susceptibility test.

DNA extracts were prepared by suspending approximately 10 mg wet bacterial cells in 100 µl of sterile distilled water and subsequent heating at 80°C to 100°C for 30 min to kill and lyse the cells [67]. Cell debris were removed by centrifugation at 13,000 g for 2 min. The lysates were stored at −20°C until further use.

### PCR amplification

Two microliters of lysates obtained from the cultured *M. tuberculosis* strains were added to 13 µl of PCR mixture, which included 1.5 µl dNTP mix (2 mM each), 1.5 µl 10×Buffer (including 25 mM MgCl_2_), 3 µl 5 M Betain, 1.0 µl primers (a 10 µM mix of each of the lower and upper primers), 0.5 µl DNA polymerase (5 U/µl), and 3 µl distilled water, to obtain a 15 µl total volume. The PCR amplification cycles consisted of: 3 min 94°C for DNA denaturation; 35 cycles: 30 seconds at 94°C for DNA denaturation, 1 min at 62°C for primer annealing and 30 second at 70°C for primer extension; following by a last cycle of 10 min at 72°C for primer extension. PCR products were analyzed by electrophoresis on a 2% agarose gel.

### Variable Number of Tandem Repeat (VNTR) typing

PCR amplification of 21 VNTR loci and electrophoresis of products on agarose gels was carried out as described in a previous report [68]. The VNTR15_China_ assay comprised the following markers: ETR-A, ETR-B, ETR-C, ETR-D (MIRU04), ETR-E (MIRU31), MIRU10, MIRU16, MIRU23, MIRU26, MIRU27, MIRU39, MIRU40, Mtub21, Mtub30, Mtub39. To compare the informativity of the VNTR21_Orsay_ and the VNTR15_China_ assays a selection of 98 isolates was made in five provinces in which the prevalence of Beijing genotype was 56 to 94%. The 15 VNTRs are present in the VNTR24 scheme described by Supply et al. [11]. Comparison with the gold standard IS6110-RFLP genotyping method was not performed.

### Region of Deletion analysis

The regions of deletion (RD) 105 and 181 were investigated using primers localized on both side of the regions as described by Tsolaki et al. [69]. In addition primers were designed that were localized inside RD181: RD181Int_L 5′ TAACAGCAGTGGGACCAAGC 3′ and RD181Int_R 5′ GACTGCCGGTCTTAGTCTGC 3′. TbD1 was investigated using primers described by Brosch et al. [15].

### Data management and analyses

Gel images were analyzed using the BioNumerics software package (version 6.5; Applied-Maths, Sint-Martens-Latem, Belgium) as previously described [68], [70]. The number of repeats in each allele was deduced from the amplicon size. The resulting data were analyzed with BioNumerics as a character data set. Clustering analysis was done using the categorical parameter and the unweighted pair group method with arithmetic averages coefficient. The minimum spanning tree [71] was constructed with the following options: (i) in case of equivalent solutions in terms of calculated distances, the selected tree was the one containing the highest number of links between genotypes differing at only one locus (“Highest number of single locus variants” option); (ii) the creation of hypothetical types (missing links) reducing the total length of the tree was allowed. Hunter-Gaston Index (HGDI) [72] is calculated by the equation:
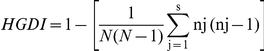
For comparison we used data from the MIRU-VNTR*plus* database at http://www.miru-vntrplus.org/ [73], and the VNTR profiles of 616 isolates genotyped in the Institute of Genetics and Microbiology, Paris Sud University http://mlva.u-psud.fr. Part of these strains were previously described [10], [74], [75]. The remaining samples were isolated by M. Fabre and C. Soler in Percy hospital as part of TB surveillance in several African and Asian countries (publication in preparation).

### Nomenclature of superfamilies/lineages

The nomenclature of lineages currently used for describing the *M. tuberculosis* complex reflects the different methods which have been applied to characterize the complex over decades of investigations. We have used in the present study the classification of Comas et al. which takes advantage of large sequence polymorphisms and sequence analysis to define 6 main lineages [12], [17]. We also refer to superfamilies which are based upon spoligotyping as defined by Filiol et al. [19]: the ancestrally branched East Africa and India (EAI) clade, the Central Asia (CAS) clade, the Beijing family, the Latin America and Mediterranean (LAM) family, the West-African 1 (AFRI1) and West-African 2 (AFRI2) clades. Lineages 1, 2, 3, 5 and 6 described by Comas et al. include respectively EAI, Beijing, CAS, AFRI2 and AFRI1. Lineage 4, also called Euro-american includes the LAM, Haarlem, T, X and other families characterized by deletion of spacers S33 to S36. Animal-adapted species of MTBC are clustered in a separate lineage called *M. bovis* phylogenetically closest to lineage 6 strains.

Beijing family strains are subdivided according to the presence/absence of RD181 into ancient and modern Beijing isolates.

## Supporting Information

Table S1 Comparison between MLVA21_Orsay_ and MLVA15_China_. The table indicates the diversity index and confidence interval for the 22 VNTR loci as estimated in the 98 isolates test panel. It also indicates which loci are included in MLVA21_Orsay_ and MLVA15_China_.
(DOC)

Table S2 MLVA15_China_ profile of 1586 Chinese isolates. The table provides the MLVA15_China_ typing data in 1586 chinese isolates. The H37Rv profile as deduced from *in silico* analysis of refseq sequence NC_000962 is also indicated for data compatibility purposes.
(XLS)

Table S3 MLVA15_China_ profile of 616 isolates from the Orsay collection. The table provides the MLVA15_China_ typing data from a representative extract of the Orsay database. The H37Rv profile as deduced from *in silico* analysis of refseq sequence NC_000962 is also indicated for data compatibility purposes.
(XLS)

Figure S1 Clustering analysis of data from 98 isolates genotyped with A) VNTR21_Orsay_ or B) VNTR15_China_ scheme. The three larger clusters defined with a cut-off value of 60% are shown with colours.
(PDF)

Figure S2 Clustering analysis of data from 900 isolates including 98 Chinese isolates. Red, 98 isolates from China. Blue, 616 isolates from the Orsay collection. Green, 186 isolates from the MIRU-VNTR*plus* database.
(PDF)

Figure S3 Minimum spanning tree for strains isolated from Xinjiang autonomous region. Isolates coloured in dark green and circled represent a specific subgroup of lineage 4.
(PDF)

Figure S4 Minimum spanning tree for strains isolated from Guangxi autonomous region. Candidate ancestrally branched isolates (dark purple) are circled.
(PDF)
