# Supplementary material for: Investigation on Mycobacterium tuberculosis Diversity in China and the Origin of the Beijing Clade
Source: PLoS One. 2011 Dec 29;6(12):e29190. doi: 10.1371/journal.pone.0029190 (PMC3248407; doi:10.1371/journal.pone.0029190)
Supplement: Table S1 — Comparison between MLVA21Orsay and MLVA15China. The table indicates the diversity index and confidence interval for the 22 VNTR loci as estimated in the 98 isolates test panel. It also indicates which loci are included in MLVA21Orsay and MLVA15China. (DOC) [file pone.0029190.s001.doc]

**Table S1**

| locus | total n=98 | standard deviation | MLVA21_Orsay_ | MLVA15_China_ |
| --- | --- | --- | --- | --- |
| ETRA-2165 | 0.3516 | [0.2476,0.4555] | X | X |
| ETRB-2461 | 0.1702 | [0.0728,0.2676] | X | X |
| ETRC-0577 | 0.0987 | [0.0174,0.1800] | X | X |
| ETRD-0580 | 0.2935 | [0.1771,0.4099] | X | X |
| ETRE-3192 | 0.4746 | [0.3652,0.5841] | X | X |
| MIRU02-0154 | 0.0204 | [0.0000,0.0602] | X |  |
| MIRU10-0959 | 0.4324 | [0.3310,0.5337] | X | X |
| MIRU16-1644 | 0.3217 | [0.2067,0.4366] | X | X |
| MIRU23-2531 | 0.2527 | [0.1464,0.3590] | X | X |
| MIRU26-2996 | 0.6070 | [0.5053,0.7087] |  | X |
| MIRU27-3006 | 0.2527 | [0.1464,0.3590] | X | X |
| MIRU39-4348 | 0.4288 | [0.3387,0.5189] | X | X |
| MIRU40-0802 | 0.4559 | [0.3575,0.5544] | X | X |
| Mtub01-0024 | 0.3444 | [0.2461,0.4427] | X |  |
| Mtub02-0079 | 0.5748 | [0.4677,0.6819] | X |  |
| Mtub12-1121 | 0.0600 | [0.0000,0.1253] | X |  |
| Mtub21-1955 | 0.6884 | [0.6223,0.7545] | X | X |
| Mtub29-2347 | 0.0406 | [0.0000,0.0960] | X |  |
| Mtub30-2401 | 0.3629 | [0.2721,0.4538] | X | X |
| Mtub38-3663 | 0.6186 | [0.5611,0.6760] | X |  |
| Mtub39-3690 | 0.3793 | [0.2655,0.4932] | X | X |
| Qub11a-2163 | 0.7153 | [0.6414,0.7892] | X |  |
| Total |  |  | 0.9960 [0.9925,0.9995] | 0.9752 [0.9547,0.9957] |
