# Supplementary figures and images for: Investigation on Mycobacterium tuberculosis Diversity in China and the Origin of the Beijing Clade
Source: PLoS One. 2011 Dec 29;6(12):e29190. doi: 10.1371/journal.pone.0029190 (PMC3248407; doi:10.1371/journal.pone.0029190)

Figure S1

cut-off value of 60%

A

MLVA\_19

85 genotypes

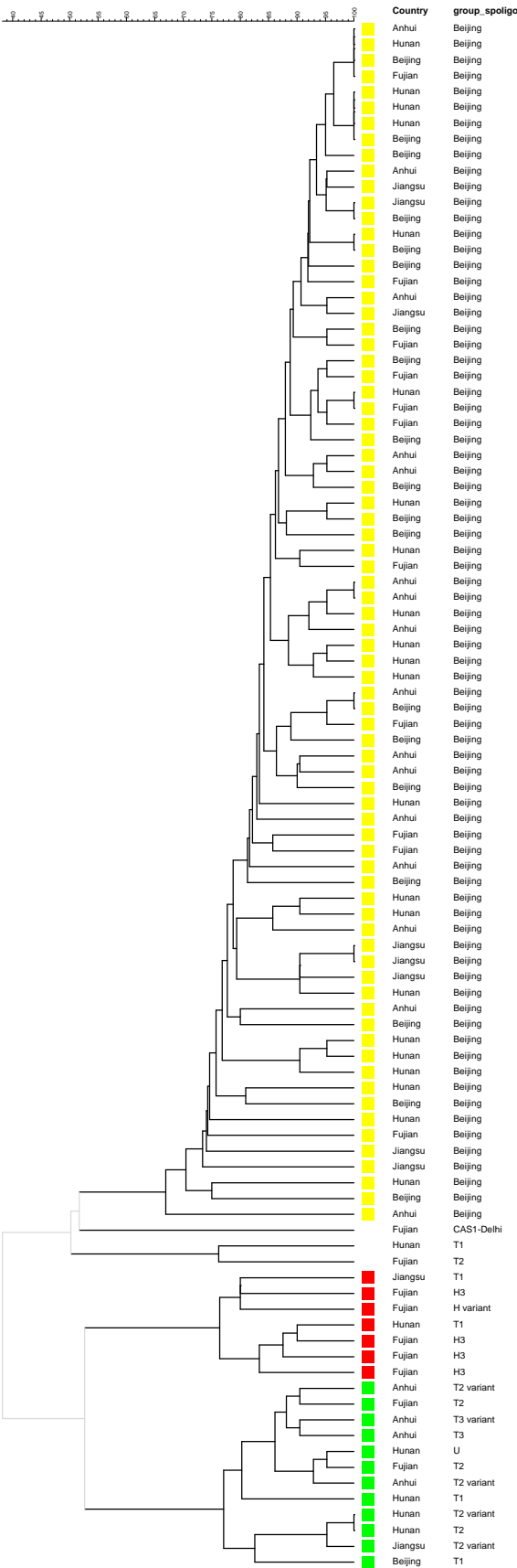

B

MLVA\_15

74 genotypes

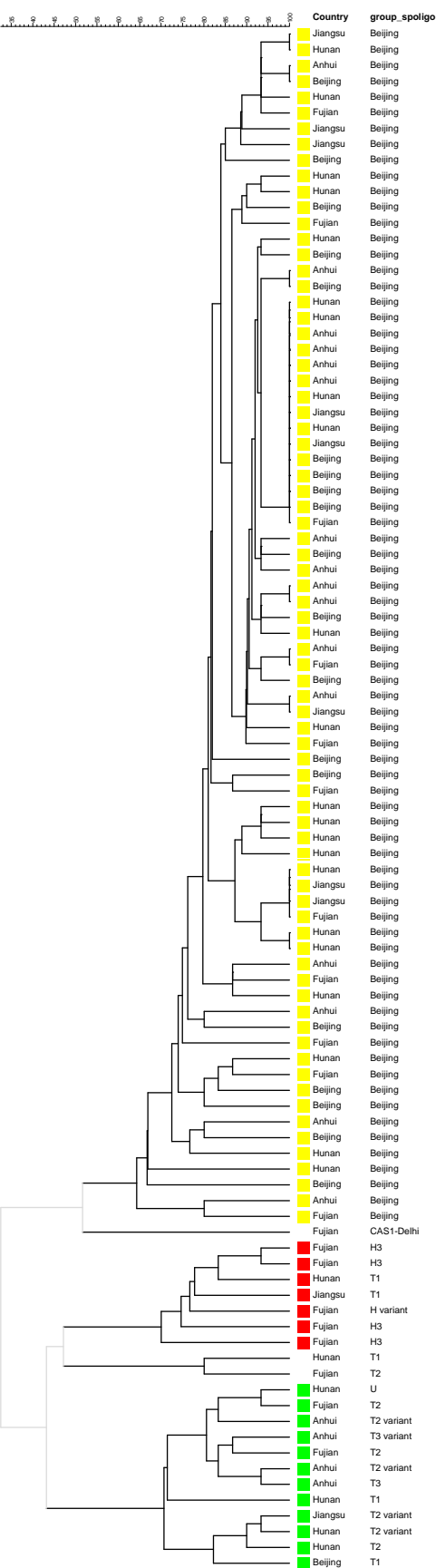

Supplement: Figure S1 — Clustering analysis of data from 98 isolates genotyped with A) VNTR21Orsay or B) VNTR15China scheme. The three larger clusters defined with a cut-off value of 60% are shown with colours. (PDF) [file pone.0029190.s004.pdf]

Figure S2

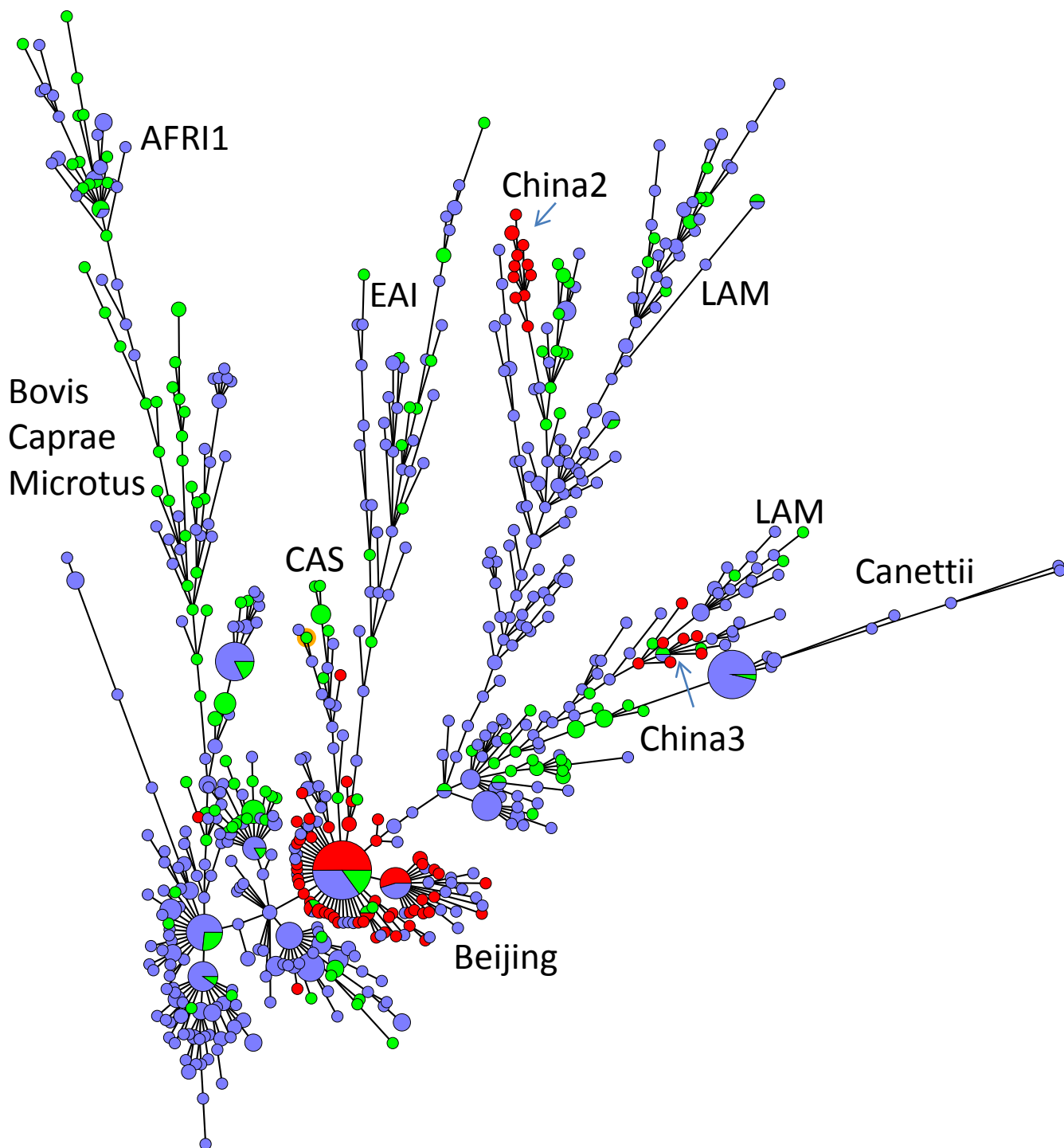

900 entries

Supplement: Figure S2 — Clustering analysis of data from 900 isolates including 98 Chinese isolates. Red, 98 isolates from China. Blue, 616 isolates from the Orsay collection. Green, 186 isolates from the MIRU-VNTRplus database. (PDF) [file pone.0029190.s005.pdf]

Figure S3

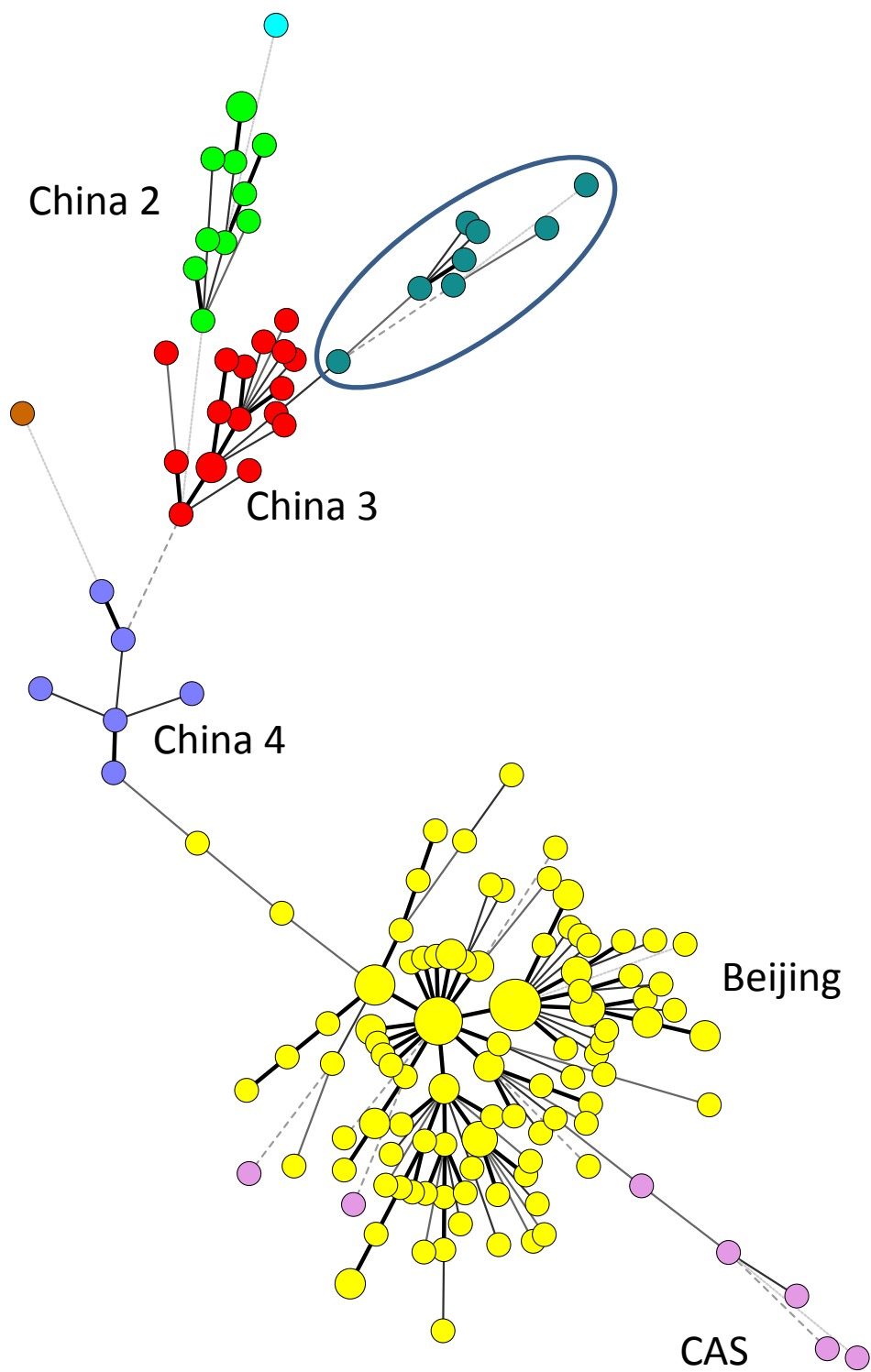

Supplement: Figure S3 — Minimum spanning tree for strains isolated from Xinjiang autonomous region. Isolates coloured in dark green and circled represent a specific subgroup of lineage 4. (PDF) [file pone.0029190.s006.pdf]

Figure S4

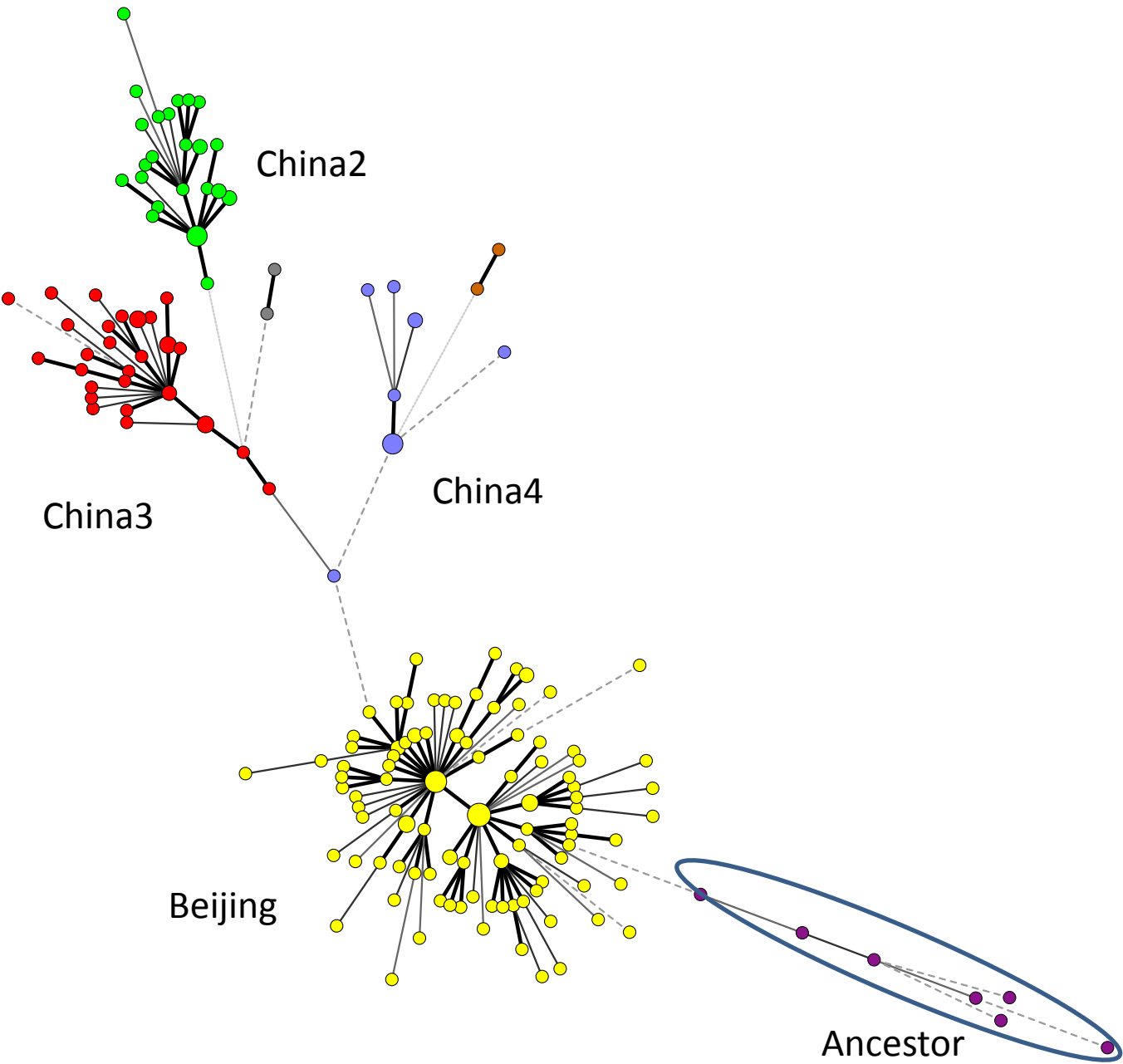

Supplement: Figure S4 — Minimum spanning tree for strains isolated from Guangxi autonomous region. Candidate ancestrally branched isolates (dark purple) are circled. (PDF) [file pone.0029190.s007.pdf]
